# 

**Published:** 1882-12

**Authors:** 


### CONTENTS.

***MISCELLANEOUS.*** PAGE

The Past and the Future. Editorial, . . . . . . . . 473

The Law of Cure, the Guide. Carroll Dunham (extract), . . . 473

The London Lancet Describes the Homœopathic Physician (extract), . 474

Plagiarism. Ad. Lippe, M. D., . . . . . . . . 474

Better After Sleep. C. Hering (extract), . . . . . . 477

Bœnninghausen's Experience with High Potencies, . . . . 477

Sulphur and Malaria (extract), . . . . . . . . 479

Motion as a Cause of Aggravation. Dr. C. von Bœnninghausen, . . 479

A Rare Symptom. C. Hering, . . . . . . . . 485

Checking Pulmonary Hemorrhage (extract), . . . . . . 485

Pure Homœopathy, Progressive Homœopathy, and the True Homœopathician. Daniel W. Clausen, M. D., . . . . . . 485

The Central N. Y. Hom. Med. Society. C. P. Jennings, M. D., Sect'y, . 493

Hypodermic Morphia, . . . . . . . . . . 499

"False Doctrin," . . . . . . . . . . . 499

The Repetition of the Dose. C. Lippe, M. D., . . . . . 500

Diphtheria: A Disease of the Liver (extract), . . . . . 505

***CLINICAL BUREAU.***

A Case of Perineal Abscess. E. Carleton, M. D., . . . . . 502
Berberis in Colic with Inflammatory Stricture. G. N. Birgham, M. D., 506
Rheumatism—Lac. Caninum. C. W. Butler, M. D., . . . . 507

***BOOK NOTICES.***

Transactions of the Minnesota State Homœpathic Institute. Drs. Camp, Leonard & Crary, Publishing Committee, . . . . . 508

Ockford: A Hand-Book of Homœopathic Practice. Duncan Bros., . 508

Campbell: Helps to Hear. Duncan Bros., . . . . . . 508

***APPENDIX.***

Cough Symptoms, . . . . . . . . . . . 41–44
Hæmorrhoids. Dr. Guernsey, . . . . . . . . 17–25

